# Stretching the boundaries of medical education A case of medical college embracing humanities and social sciences in medical education

**DOI:** 10.12669/pjms.324.10237

**Published:** 2016

**Authors:** Kulsoom Ghias, Kausar S Khan, Rukhsana Ali, Shireen Azfar, Rashida Ahmed

**Affiliations:** 1 Kulsoom Ghias, PhD. Department of Biological and Biomedical Sciences, Aga Khan University, Karachi, Pakistan; 2 Kausar S Khan, MA. Department of Community Health Sciences, Aga Khan University, Karachi, Pakistan; 3 Rukhsana Ali, MA. Curriculum Office, Aga Khan University, Karachi, Pakistan; 4 Shireen Azfar, MA. Office of the President, Aga Khan University, Karachi, Pakistan; 5 Rashida Ahmed, MBBS, FCPS, MHPE. Department of Pathology and Laboratory Medicine, Aga Khan University, Karachi, Pakistan

**Keywords:** Medical education, Humanities, Social sciences, Curriculum

## Abstract

**Objective:**

Aga Khan University, a private medical college, had a vision of producing physicians who are not only scientifically competent, but also socially sensitive, the latter by exposure of medical students to a broad-based curriculum. The objective of this study was to identify the genesis of broad-based education and its integration into the undergraduate medical education program as the Humanities and Social Sciences (HASS) course.

**Methods:**

A qualitative methodology was used for this study. Sources of data included document review and in-depth key informant interviews. Nvivo software was utilized to extract themes.

**Results:**

The study revealed the process of operationalization of the institutional vision to produce competent and culturally sensitive physicians. The delay in the establishment of the Faculty of Arts and Sciences, which was expected to take a lead role in the delivery of a broad-based education, led to the development of an innovative HASS course in the medical curriculum. The study also identified availability of faculty and resistance from students as challenges faced in the implementation and evolution of HASS.

**Conclusions:**

The description of the journey and viability of integration of HASS into the medical curriculum offers a model to medical colleges seeking ways to produce socially sensitive physicians.

## INTRODUCTION

Conventionally, medical education has focused on building scientific competencies for treating diseases. This focus inadvertently renders the patient secondary, giving rise to the criticism that instead of treating the patient, only the disease is treated.^1^ This ethical concern, a significant landmark in clinical care, arose from within the health care community and underlies the idea of a ‘good’ or ‘ethical’ physician, concerned about the patient and not just the disease. What constitutes a ‘good physician’ may be intuitive, but it has been formally studied and documented in the literature. In 1988, the World Federation of Medical Education declared that a physician has to have two characteristics – to be scientifically competent, and ethical.^2^,^3^

While scientific competency can be achieved through medical curricula, developing an ethical and therefore, good physician, requires additional attention. Many institutions have dedicated bioethics curricula.^4^ However, a discourse on the importance of a broader education, through the study of humanities and social sciences (HASS) or liberal arts, to develop a holistic medical graduate is also found in the literature.^5^-^7^

In Pakistan, students enter medical college upon completion of secondary education with a focus on required science subjects. The regulatory body, Pakistan Medical and Dental Council (PMDC), does not require that students entering medical colleges should have a broad-based education or that the medical curriculum should provide it. However, PMDC requires the inclusion of Islamic and Pakistan studies.^8^ The Aga Khan University (AKU) Medical College, a private medical college established in Karachi in 1983, follows PMDC requirements, but has additional curricular requirements that include humanities and social sciences (HASS) courses in the medical curriculum.

The objective of this study was to understand why AKU Medical College imposes this additional curricular requirement of HASS. Specifically, we investigated the genesis of the idea of a broad-based education and how this is aligned with the institutional vision of producing scientifically competent and socially sensitive physicians. The journey of integration of a broader education into the undergraduate medical education program as the Humanities and Social Sciences (HASS) course was delineated.

## METHODS

A qualitative study design approved by the Aga Khan University Ethical Review Committee was used to achieve the objective of the study. Two sources of data were used; document review and in-depth interviews. Documents reviewed included the Chancellor’s Commission Report, PMDC curriculum, internal course documents, minutes of internal committee meetings, and student feedback forms.

Interviews included those of key persons involved in the inception phase of AKU and operationalization of the institution’s vision (3 key informants), involved in the early implementation of humanities and social sciences in medical education curriculum (1 key informant), and in the evolution of the curriculum (2 key informants). The semi-structured interviews were transcribed. Analysis was done through Nvivo version 2.6 and different themes were extracted. Findings from the interview were triangulated with the content of the documents studies.

## RESULTS

The study showed that three decades since the inception of AKU can be discerned as three distinct periods with regards to HASS, specifically (i) conceptualization and operationalization of the vision of the institution; (ii) inception of HASS; and (iii) evolution of the HASS curriculum. The important themes identified within these periods are (i) broad-based education, (ii) teaching of Islamic and Pakistan studies, (iii) challenges in implementing of broad-based education. Table-I shows the triangulation of representative data between interview findings and document review in relation to the identified themes.

**Table-I:** Data triangulation of representative data for each theme.

Themes	Key informant comments	Excerpts from document(s) reviewed
Broad-based education	“Broad-based education as the discipline of mind that acquires, evaluates and communicates knowledge, and which needs to be developed…” “This curriculum should be broadened, now that we are looking at the Faculty of Arts and Sciences, we should have two years over there and then come here [Medical College] for four years.”	*Chancellor’s Commission Report:* “It will be a university spread broadly both geographically and over the fields of both pure and applied knowledge from sciences to humanities.” “We recommend that the recent proposals for giving a more general and liberal education to students in the Faculty be carried forward either in the form of a preliminary year or otherwise.”
Teaching of Islamic and Pakistan Studies	“In 1982, [we were] struggling with this whole business of Islamic and Pakistan Studies, which the Ministry of Education… demanded that nobody can get a degree without… So what do we do with this Islamic and Pakistan Studies?” “Islamic Studies at university level is taught in the framework of humanities and social sciences…renowned experts in various areas [came] to talk to the [medical] students.”	*From PMDC curriculum document:* “Sessions on Islamiyat and Pakistan Studies will be included in Years 1 and 2 [of medical curriculum].” *From initial Islamic and Pakistan Studies internal course documents:* “[Select] Session topics: 1. Religion and Human Civilization 2. Fundamentalism, Liberalism and Modernism 3. Introduction to Muslim Philosophy… …at least 20% of lectures [to be] given by scholars from outside”
Challenges in implementing broad-based education	“Conceptualization was not a problem. The actual implementation of [broad-based education], overseeing it, managing the whole process, that was another story altogether…”	*From UGME Curriculum Committee minutes:* “…the course had to be conceptualized…without the help of any dedicated HASS team.” “…we are experimenting with HASS courses…it is [still] based on the convenience and availability of the highly qualified faculty.” Student feedback: “Initially, I thought the HASS courses would be boring…”

### Conceptualization and operationalization of vision

The Chancellor of AKU, His Highness the Aga Khan IV, founded the institution with a vision to create a difference in society that was struggling with poor health indicators despite the fact that sixteen public medical colleges were graduating physicians at the time. The institutional vision explicitly went beyond the medical model of health and identified the need to understand the socio-cultural contexts in which patients live.

The desired attributes and outcomes of AKU graduates were deliberated at length with support of experts from Harvard University and Johns Hopkins University. These deliberations led to the understanding of “broad-based education as the discipline of mind that acquires, evaluates and communicates knowledge, and which needs to be developed [in AKU students]” (Key informant).

The Faculty of Health Sciences, which included a medical college, was planned to produce scientifically competent and ethical doctors. Another stand-alone Faculty of Arts and Sciences (FAS) was envisioned for liberal arts education. Document review triangulated with key informant interviews revealed that while the medical college became functional in 1983, plans for FAS and the idea of a broad-based education were presented in 1994 in a report titled the Chancellor’s Commission Report. FAS was envisaged to provide a foundational base by offering two years of liberal arts education prior to entering the undergraduate medical education (UGME) program.

### Inception of HASS

The study revealed that while the vision was clear, the medical educationists faced the challenge of enabling medical students to learn about communities and the sociocultural environment that shape their living conditions and health behaviour.

This was achieved by adopting a community-based medical education approach through the Department of Community Health Sciences.^9^ This included a direct interaction with communities to bolster students’ understanding of community and their dynamics. Bioethics was also gradually included in the curriculum of undergraduate medical students, but there was still a gap in the desired broad-based education. The envisaged support from FAS was not available, as its implementation plans were repeatedly delayed by factors beyond the control of the institution. This left the medical college with the challenge of integrating HASS in UGME.

Key informant interviews revealed that in the absence of FAS, the UGME Curriculum Committee took on this challenge, appreciating the importance of HASS for the holistic development of medical students. Strategically, this challenge was addressed by capitalizing on the government requirement of teaching two mandatory subjects, Islamic and Pakistan Studies (IPS), in all professional programs and using it as an opportunity to integrate elements of a broad based education. A scholar who understood the requirement of a broad-based education planned and taught the two mandatory subjects, modifying the curriculum to partially fulfill the institutional aims. Specifically, Islamic history was made relevant through critical analysis of past events and correlation to current issues. According to the scholar, who was a key informant in this study, student interest was engaged by inviting visiting scholars and experts in various fields who contributed to the teaching of the two mandatory subjects.

While the institution started its HASS journey with the curriculum providing a more critical understanding of Islam and Pakistan, other HASS subjects that FAS could have provided were not introduced to the students at this stage.

### Evolution of HASS

While the two mandatory subjects were being taught in innovative ways, deliberations continued to identify the possible contents of a formal HASS curriculum that could serve as a “foundational course for medicine” (Key informant). In 2007, the first iteration included one-off lectures on various HASS topics in UGME Year 1, utilizing 1-2 hours a week. Four weeks were allocated at the end of Year 1 for electives in any HASS subject. A limited selection of elective courses was offered at AKU, such as Photography and English Literature. Students, however, had the flexibility of completing the HASS elective requirement at any reputable institution. In 2011, under the aegis of a dedicated HASS curriculum sub-committee, a two-week course was designed and implemented focusing on HASS subjects such as literature and art through the lens of gender and citizenship. Eventually, HASS evolved into a dedicated 6-week course at the beginning of Year 1. To date (i.e. the 2015 – 2016 academic year), the HASS curriculum has evolved to include two required courses titled Religions of Pakistan and Silent Stories of Pakistan to satisfy the PMDC requirements for Islamic and Pakistan Studies, respectively and numerous electives (Table-II). Over a five year period, the HASS curriculum has been taught by over 45 adjunct and visiting faculty using a variety of pedagogical approaches.

**Table-II:** HASS electives offered since the implementation of formal 6-week module.

Academic year	Electives	
2011 - 2012	Introduction Social Sciences	Introduction to Documentary Film-making
	English Literature	Urdu Literature
	Spanish	Arabic – Quranic Grammar
	Persian	Music of South Asia
	Photography	Anatomy of Art
2012 - 2013	Introduction to Philosophy	Introduction to Psychology
	Introduction to Documentary Film-making	Literature in English
	Urdu Literature	Creative Writing – Non-Fiction
	Persian Language	Arabic – Quranic Grammar
	Arabic-Conversational	Spanish
	Theatre	Music of South Asia
	Photography	Physiology of Art
	Sketching	Oil Painting
	Sculpture	Calligraphy
2013 - 2014	Introduction to Philosophy	Measure for Measure – An Introduction to Law
	Literature in English	Creative Writing – Non-Fiction
	Historical Problem, Historical Solutions – A Brief History of Mathematic Media & Communication Studies	Art, Politics and the Performing Body
	Introduction to Documentary Film-Making	Take 2 – Hollywood on Morality
	Persian Language	Chinese Language
	Theatre	Fine Arts: Drawing & Painting
	Music – Mysteries and Meditation	Photography
2014 - 2015	Philosophical Inquiry	Measure for Measure – An Introduction to Law
	Literature in English	Urdu Literature
	Creative Writing – Non-Fiction	Exploration in Contemporary Art and New Media
	Historical Problem, Historical Solutions – A Brief History of Mathematic	
	Life is Comedy	Spanish Language
	Persian Language	German Language
	Chinese Language	Introduction to Documentary Film-Making
	Photography	Theatre
	Music – Mysteries and Meditation	Art: Drawing & Painting
	Art History	
2015 - 2016	Philosophical Inquiry	Measure for Measure – An Introduction to Law
	Literature in English	Urdu Literature
	Creative Writing – Non-Fiction	An Introduction to Anthropology
	Spanish Language	Persian Language
	Chinese Language	Introduction to Documentary Film-Making
	Photography	Theatre
	Music – A Healer’s Art	Art: Drawing & Painting

This study has shown that implementation of the extended 6-week HASS module has not been free of challenges. One major issue has been the identification of faculty to teach HASS courses. As a medical college, AKU does not have any full-time qualified faculty to teach humanities and social sciences. Faculty from outside AKU have to be identified and vetted. Retention of the same faculty from year to year has been a challenge. The physical infrastructure of the medical college campus is also not ideally suited to the study of subjects such as Music, Drawing, Painting, Sculpting and Theatre. Finally, students bring an internal barrier to HASS as it is a subject alien to their understanding of medical education. Coming from a background where humanities and social sciences are traditionally not considered relevant to medical students, many students are initially very critical of the HASS module. There is a lack of appreciation for the importance of studying humanities in a professional medical program. This barrier is overcome once the students go through the HASS courses, which routinely receives largely positive feedback through structured evaluation forms, examples of which are given below:

“I like the fact that future doctors are made to study humanities and social sciences so they can think about stuff other than medicines, patients and hospitals. Initially I thought the HASS courses would be boring, but now I realize that life is not only about science and technicalities, it’s about you and me.”

“[HASS] had a great impact on me. I now know, no matter what, humanity comes first. I hope I can implement it now and in my life when I become a doctor.”

## DISCUSSION

### History of broad-based education

At the time that AKU was in the planning phase, there was no regional discourse on the need to integrate a broad-based education prior to or with the medical curriculum. However, there were examples of curricula from around the world, such as the United States where students enroll in medical education programs *after* four years of broad-based education. This was not always the case. In 19^th^ century United States, three systems of medical education were functioning – the apprenticeship model, the proprietary school model and the university model.^10^ At this time, there was no regulation of medical practice or medical education. Throughout the second half of the 19^th^ century, the American Medical Association (AMA) tried to standardize medical education. In 1904, through the Council of Medical Education, the AMA began to promote the restructuring of the United States’ medical education via standardization of preliminary educational requirements for entry into medical school.^11^ In 1910, Flexner’s Report was published that helped to consolidate this movement. The report recommended that: a) a high school education and at least two years of college level or university science should be the minimum admission standards; b) medical schools should be four years in duration - two years of basic sciences and two years clinical; and c) the proprietary schools should be closed down or merged into the universities.^12^ By 1930, the pre-medical science requirements were set and followed for nearly half a century. In 1984, the Physicians for the 21^st^ Century Report recommended the baccalaureate preparation for medicine and modifying medical school admission requirements. The report pointed out that the “narrow and extremely focused” requirement for medical school admission resulted in an unbalanced college experience that excluded the possibility of a broad liberal arts education. The report recommended “that all physicians should not only acquire and sustain clinical expertise, skills and knowledge, but also hone and apply humanistic values and attitudes common to a profession dedicated to caring and healing”.^13^ Meanwhile a discourse on the requirement of a broad education can be seen in the literature for UK-based institutions since the 1980s,^14^ where medical students enter a professional programme after secondary education with some science requirements.

### Beyond teaching of Islamic and Pakistan studies

In Pakistan, the western style medical education that was brought into the pre-partitioned sub-continent in the 19^th^ century continues to date. Students enter medical college at the average age of 18 years upon completion of secondary education with a focus on required science subjects. As mandated by the government, PMDC requires the teaching of Islamic and Pakistan Studies in medical colleges. However, it does not have any pre- or post-admission requirements of a broad-based education, implying that scientific knowledge is central to medical education, and thereby rendering to the periphery essential knowledge included in humanities and social sciences. In the context of Pakistan where unethical practices are common and there is lack of governance and accountability,^15^ a broad-based education that encompasses academic disciplines beyond religious and regional studies is particularly critical in order to graduate good physicians.

### Overcoming challenges in incorporating broad-based education in medical curricula

Creating an environment where medicine and HASS have equal standing and contribution has been identified as a challenge for medical humanities programs in other studies.^16^ AKU Medical College now has a model in the form of a 6-week dedicated course that integrates HASS in the medical program. Offering HASS from within the medical college exhibits commitment to the institutional vision, dispels the notion that it is non-essential “add-on” and thereby, strengthens the contribution of HASS in the making of a good physician. This idea was presented in a workshop during a medical education conference and was well-received by participants from all over Pakistan.^17^ However, some challenges pertaining to recruitment and retention of a core faculty, and finding suitable physical spaces for teaching and learning still remain.

## CONCLUSION

The founders of AKU wanted to train a new breed of competent physicians with a sound scientific base for understanding health, and also educating the students to be sensitive to people with poor health created by their social economic conditions. From its inception in 1983 AKU has been committed to including a broader education in the undergraduate medical curriculum in an effort to graduate ‘good’ physicians.

AKU already has subjects such as Bioethics, Behavioural Sciences, and Communication Skills that are included longitudinally throughout the entire five-year curriculum. While HASS has been successfully integrated in Year 1 of the undergraduate medical program at AKU, further work is required to introduce it as a longitudinal theme and address the challenges currently faced in its implementation. Additional studies are also required to substantiate that inclusion of HASS produces a more holistic and therefore, better medical practitioner. Evaluation studies are required to determine the impact of HASS on students, the overall curriculum and the learning environment.

In the meantime, this study provides a roadmap that needs to be critically examined by other institutions. A robust discourse is needed in Pakistan on the relationship between HASS and medical education. Partnerships need to be built between the disciplines of medicine and social sciences and humanities to address the health challenges faced by the people of Pakistan and other developing countries that have glaring inequalities and where the state is not responsive to the needs of the poor and marginalized. Physicians in these countries who are culturally sensitive will be better equipped to achieve more positive health indicators through ethical and patient-centered care.
